# Identification of germ cell-specific VASA and IFITM3 proteins in human ovarian endometriosis

**DOI:** 10.1186/s13048-015-0193-8

**Published:** 2015-10-07

**Authors:** Nicolas A. Fraunhoffer, Analía Meilerman Abuelafia, Inés Stella, Silvia Galliano, Marcela Barrios, Alfredo D. Vitullo

**Affiliations:** Centro de Estudios Biomédicos, Biotecnológicos, Ambientales y Diagnóstico, CEBBAD- Universidad Maimónides, Hidalgo 775, Buenos Aires, C1405BCK Argentina; Consejo Nacional de Investigaciones Científicas y Técnicas, CONICET-Argentina, Buenos Aires, Argentina; Facultad de Medicina, Universidad Maimónides, Buenos Aires, Argentina; Servicio de Anatomía Patológica, Hospital Eva Perón, Merlo, Buenos Aires Argentina

**Keywords:** Ovarian endometriosis, Ovarian stem cells, DDX4, IFITM3

## Abstract

**Background:**

Endometriosis is a gynaecological disorder that affects 6–10 % of female population. It is characterized by the presence of endometrial tissue outside the uterus, most often in the pelvic peritoneum or ovaries. Recent studies have indicated that mesenchymal endometrial stem cells might get involved in endometriosis progression. Although germ line stem cells have been proved to exist in the ovary, their involvement in ovarian endometriosis has not been investigated. In this preliminary report we aimed to identify germinal stem cell markers in ovarian endometriosis.

**Findings:**

Ten paraffin-embedded ovarian endometriosis samples were screened for germ cell-specific proteins DDX4 (VASA) and IFITM3, and its relation with stem cell marker OCT4, proliferation marker PCNA and estrogen receptor alpha (ESR1), by immunohistochemistry, immunofluorescence and PCR. DDX4 and IFITM3 proteins were expressed in isolated cells and clusters of cells in the cortical region of ovarian endometriotic cysts. DDX4 and IFITM3 co-localized in cells from endometriotic stroma, and DDX4/IFITM3-expressing cells were positive for ESR1, OCT4 and PCNA. No cells expressing neither DDX4 nor IFITM3 were detected in normal endometrial tissue.

**Conclusion:**

The identification of germ cell-specific proteins DDX4 and IFITM3 provides the first evidence of ovarian-sourced cells in ovarian endometriotic lesions and opens up new directions towards understanding the still confusing pathogenesis of endometriosis.

## Findings

### Introduction

Endometriosis is an estrogen-dependent benign chronic and multifactorial gynaecological disorder, characterized by the presence of endometrial tissue outside the uterus, most often in the pelvic peritoneum or ovaries [1, 2]. Its prevalence is estimated to be 6–10 % in the general female population and 35–50 % in patients with infertility [1]. The lesions in the ovaries are predominantly hemorrhagic and characterized by invagination and invasion of the endometrium into the ovarian cortex [3]. Endometriosis was first described by Carl von Rokitansky [4] in 1860; however, it still remains an enigmatic disease. Several theories have been proposed regarding the pathogenesis of endometriosis. The most accepted one is the “back flow” theory (retrograde menstruation) proposed by John Sampson [5] in 1925, which suggests that endometrial fragments in menstrual blood are taken over through the tubal pavilion into the peritoneal cavity initiating an endometriosis lesion. Recently, it has been postulated that endometriosis can arise from a small population of mesenchymal stem/stromal cells (eMSC) residing in the endometrial tissue [6, 7]. This point of view was supported by the identification of undifferentiating markers such as C-KIT and OCT4 into the endometrial and ectopic endometriosis tissues [8, 9].

In the last 10 years, a long-held dogma proposing that the mammalian ovary has a finite, non-renewable oocyte pool has been challenged by the finding, in the adult mouse ovary, of a small population of germ line stem cells which could differentiate into oogonia, enter meiosis and contribute to oocyte replenishment [10]. Since then, evidence claimed to be for and against the possibility of oogenesis *de novo* in the adult mammalian ovary has accumulated [11]. Whether germ line stem cells contribute to oogenesis and follicle formation *in vivo* has not been proven yet [12]. However, germ line stem cells appear to exist in the adult ovary as shown by independent investigations in human, mouse and rat [13–15]. Moreover, isolated germ line stem cells can be manipulated in vitro to give rise to offspring after transplantation [13].

Whether, and how, germ line ovarian stem cells might contribute to the establishment and progression of ovarian endometriosis has not been yet investigated. In this study, we looked for the presence of ovarian stem cells in ovarian endometriosis lesions. We identified cells expressing VASA (DDX4) and IFITM3 germ line-specific markers known to be implicated in different cellular processes including germ-cell homing and maturation [16, 17].

## Materials and methods

### Patients and samples

This study was reviewed and approved by the Research Ethics Committee of Universidad Maimónides and the Ethics Committee from Clínica San Nicolás, Buenos Aires province, Argentina. Clinical paraffin-embedded samples of endometriosis and normal endometrial tissues were obtained from the repository of the Pathology Department of the Hospital Eva Perón, Buenos Aires province, Argentina. Endometriosis samples (*n* = 10) belonged to patients aged between 18 and 34 years, and endometrial tissues (*n* = 4) from patients aged between 32 and 45 years old, undergoing hysterectomy for benign pathologies.

### Immunohistochemistry

Samples were baked overnight at 60 °C, dewaxed in xylene and hydrated through a decreasing ethanol series (100, 80, and 50 %). After 10 min in PBS, the heat-induced epitope retrieval was performed by thermic bath at 96 °C in 10 mM sodium citrate (pH 6) during 20 min. Endogenous peroxidase activity was blocked with 3 % H_2_O_2_ in methanol for 30 min, and washed twice in 0.01 % Tween 20 in PBS buffer (PBS-T) (pH 7.4). After that, sections were blocked with 1.5 % blocking serum solution (from ABC Vectastain Elite Kit, Vector Laboratories, Burlingame, USA) in PBS-T, for 30 min. Slides were incubated with the primary antibody overnight at 4 °C. Primary antibodies used included rabbit polyclonal anti-DDX4 IgG (1:200; Abcam, Cambridge, UK), and anti-IFITM3 (1:300; Abcam, Cambridge, UK). Secondary antibody used was a goat anti-rabbit IgG (ABC Vectastain Elite Kit, Vector Laboratories, Burlingame, USA) for 30 min at room temperature followed by incubation with avidin-biotinn complex (ABC Vectastain Elite Kit, Vector Laboratories, Burlingame, USA) for additional 30 min at room temperature. The reaction was visualized with 3,3'- diaminobenzidine chromogen containing nickel salt (DAB Kit, Vector laboratories, Burlingame, USA). The sections were dehydrated, and coverslipped. Images were captured and analyzed using an optic microscope (BX40, Olympus Optical Corporation, Tokyo, Japan), with a digital camera (390CU 3.2 Megapixel CCD Camera, Micrometrics, Spain).

### Immunofluorescence and co-localization

Paraffin sections were dewaxed in xylene and hydrated through decreasing ethanol. After 10 min in PBS, the heat-induced epitope retrieval was performed by thermal bath at 96 °C in 10 mM sodium citrate at pH 6 during 20 min. After that, sections were blocked with blocking serum solution (Vector Laboratories, Burlingame, USA) diluted in 1.5 % PBS-T for 30 min. Slides were incubated with the primary antibody overnight at 4 °C. Primary antibodies used included rabbit polyclonal Ig-G anti DDX4 (1:200; Abcam, Cambridge, UK), anti IFITM3 (1:300; Abcam, Cambridge, UK), anti OCT4 (1:300; Abcam, Cambridge, UK), anti PCNA (1:300; Abcam, Cambridge, UK), anti ESR1 (1:100; Santa Cruz Biotechnology Inc., Dallas, Texas, USA), and mouse monoclonal Ig-G anti CD45 (1:100; Dako, Copenhagen, Denmark). Secondary antibodies used were goat anti-rabbit FITC (1:500; Chemicon, EMD Millipore Corporation, Billerica, Massachusetts, USA), donkey anti-rabbit Alexa 555 (1:600; Invitrogen, Life Technologies Corporation, Carlsbad, California, USA), and goat anti-mouse FITC (1:300; Chemicon, EMD Millipore Corporation, Billerica, Massachusetts, USA) for 60 min at room temperature. The slides were counterstained with mounting medium for fluorescence with DAPI (H-1200; Vector Laboratories, Burlingame, USA). Negative controls were performed by omitting the primary antibody. Images were analyzed using an optic microscope (Olympus BX40) and captured with an attached digital camera (390CU 3.2 Megapixel CCD Camera, Micrometrics, Spain). Colon and ovary sections were used as positive control for IFITM3 and DDX4 antibodies, respectively.

Co-localization was performed initially in the same way as immunofluorescence, but after the first secondary antibody, the slides were rinsed twice with PBS-T for 10 min each. Then, the sections were blocked with blocking serum solution (Vector Laboratories, Burlingame, USA) diluted in 1.5 % in PBS-T for 30 min. After that, the slides were incubated with a second primary antibody overnight at 4 °C and then washed and incubated with the appropriate secondary antibody for 60 min at room temperature. Finally, slides were counterstained as indicated above.

### RNA extraction and RT-PCR

Total RNA was extracted from paraffin embedded samples using the RNeasy FFPE kit (Qiagen, Hilden, Germany). The primers used for RT-PCR were as follows: DDX4_F:5'-AGAAAGTAGTGATACTCAAGGACCA-3'; DDX4_R:5'-TGACAGAGATTAGCTTCTTCAAAAGT-3'; IFITM3_F:5'-CTGTCCAAACCTTCTTCTCTCC-3'; IFITM3_R:5'-GTAGGCGAATGCTATGAAGCC-3'. The RT-PCR was performed using QIAGEN One-step RT-PCR kit (Qiagen, Hilden, Germany).

## Results

### Identification of DDX4 and its association with hormonal, proliferation and stem cell markers

**Fig. 1:**
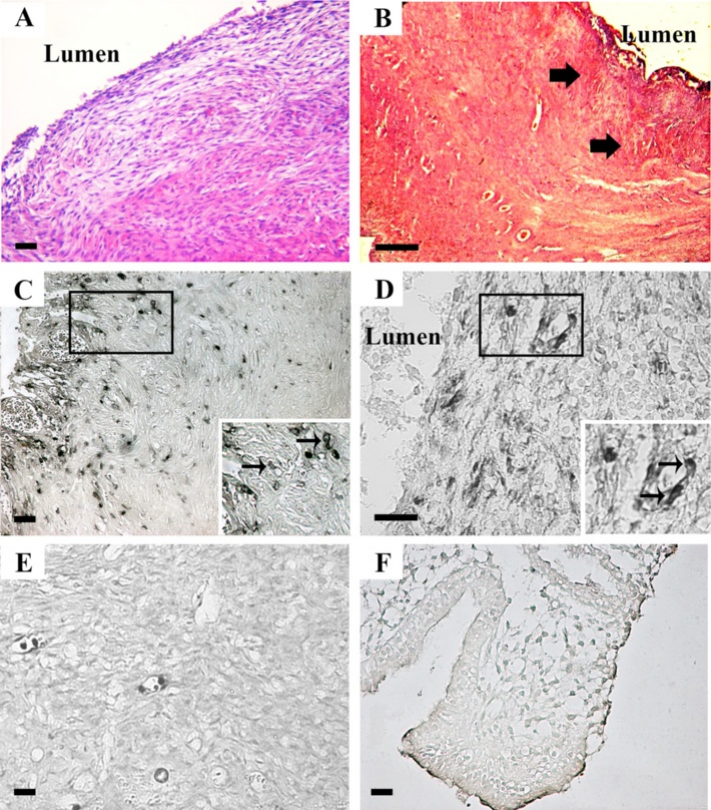
General histology and immunohistochemistry for IFITM3 and DDX4 proteins in endometriotic cysts. **a** Hematoxylin-eosin general view of the wall of the endometriotic cyst, **b**. General view of an endometriotic cyst showing large amounts of haemorrhagic debris (arrows), **c**. General view of IFITM3 immunodetection with detection of sparse cells with specific signals (inset), **d**. DDX4 immunohistochemistry also enabled to detect dispersed cells showing positive reaction (inset), **e**. Normal endometrial tissue showing no positive cells for IFITM3, and **f**. Normal endometrial tissue negative for DDX4 immunohistochemistry. Bar = 50 μm

All samples were first inspected for general histology by hematoxylin-eosin (HE) staining and subjected to exploratory immunohistochemistry (IHC) for DDX4 and IFITM3 markers (Fig. 1). Although in some cases a clean HE was observed (Fig. 1a), most of the samples showed important amounts of hemorrhagic debris (Fig. 1b) yielding unspecific precipitates in IHC (Fig. 1c, d). However, a thorough inspection led to identify sparse cells displaying suspected specific signals both for IFITM3 (Fig. 1c) and DDX4 (Fig. 1d) mostly located near the borders of the endometriotic cyst. No cells indicating a possible specific signal were detected in control endometrial tissue both for IFITM3 and DDX4 proteins (Fig. 1e, f).

**Fig. 2:**
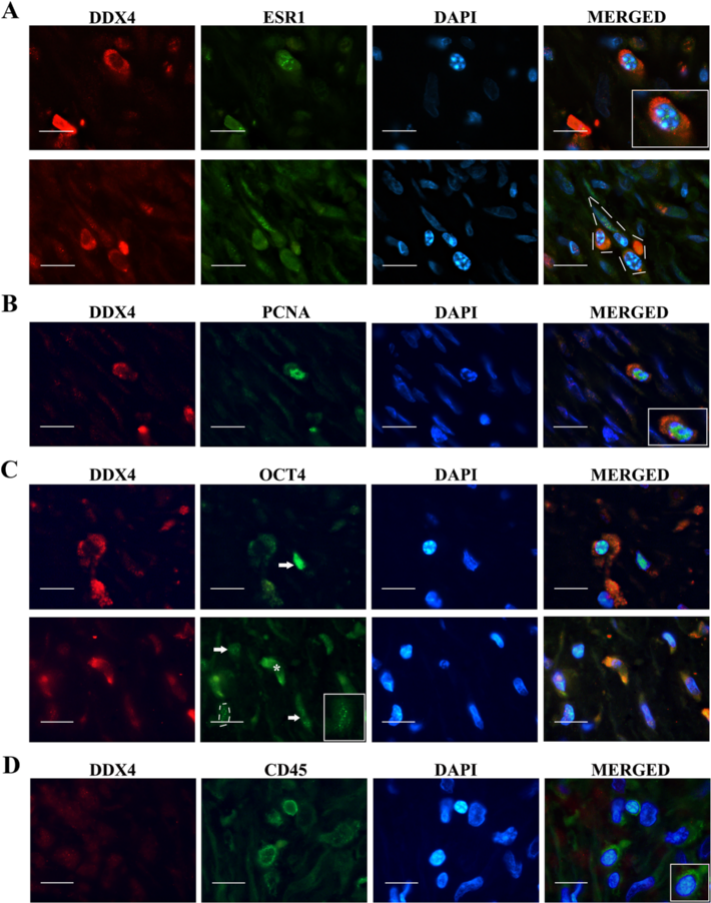
Immunofluoresecent detection of DDX4 in ovarian endometriosis lesions and its relation to hormonal, stem cell and proliferation markers. **a**. Co-expression of DDX4 and ESR1. DDX4 was detected in cytoplasm of isolated cells and clusters of cells (dashed line). ESR1 was observed in the nucleus of the cells with positive cytoplasmatic staining for DDX4 (inset). **b** Co-expression of DDX4 and PCNA. DDX4 was observed in cytoplasm of cells with strong positive nuclear staining for PCNA (inset). **c** Co-expression of DDX4 and OCT4. OCT4 was mainly nuclear (arrows), but it also was observed in the cytoplasm (asterisk). The inset shows a detail of nuclear staining (dashed line). **d** CD45-positive cells did not show signal for DDX4 protein. Bar = 50 μm

**Fig. 3:**
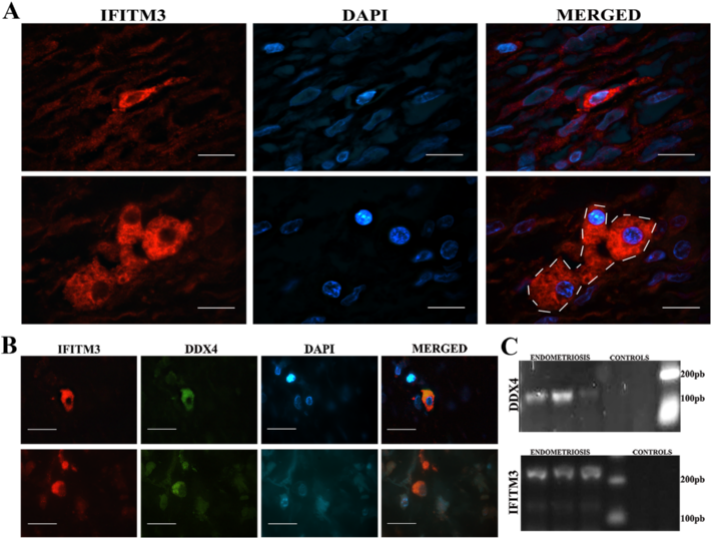
Expression of IFITM3 in ovarian endometriosis lesions and RT-PCR for DDX4 and IFITM3. **a** IFITM3 was detected in cytoplasm of isolated cells and in clusters of cells (dashed line). **b** DDX4 and IFITM3 proteins co-localized in the cytoplasm in the same cells in endometriosis lesions. **c** DDX4 and IFITM3 were detected through RT-PCR in ovarian endometriosis. Endometrial tissue was negative. Bar = 50 μm

All samples were then thoroughly analyzed by immunofluorescence. In all endometriotic samples, DDX4-positive cytoplasmic staining in stromal cells, isolated or in clusters, were undoubtedly detected (Fig. 2a). No DDX4-positive cells were detected in the 4 normal endometrial tissues inspected. Although not quantified, positive cells were low in numbers; however, samples from younger patients seemed to display higher numbers since positive cells were more easily found. PCR-analysis confirmed DDX4-mRNA expression in endometriotic samples (Fig. 3c).

We further studied the relationship between DDX4 and the characteristic hyper-estrogenic hormonal environment of this disease analyzing the co-expression of DDX4 and ESR1. In all pathological samples, we observed ESR1 staining in the nucleus of DDX4-positive cells (Fig. 2a). Additionally, DDX4-expressing cells were positive for the proliferating marker PCNA (Fig. 2b) and, some of them, also showed a weak OCT4 cytoplasmic expression (Fig. 2c). On the other hand, cells expressing CD45 lymphocyte marker did not show signals for DDX4 (Fig. 2d).

### Identification of IFITM3 and its association with DDX4

A homogeneous cytoplasm distribution of IFITM3 immunolabelling was found in isolated cells and clusters of cells in all endometriosis samples (Fig. 3a). Moreover, IFITM3 co-localized with DDX4 (Fig. 3b). No positive IFITM3 immunostaining was detected in normal endometrial tissue. The expression of IFITM3 in ovarian endometriosis lesions was confirmed by RT-PCR (Fig. 3c) and normal endometrial tissues were negative for IFITM3-mRNA.

## Discussion

We identified DDX4 and IFITM3 proteins in isolated and clusters of cells in ovarian endometriosis lesions. DDX4 and IFITM3 are essential proteins for homing and maturation of germ cells [18, 19] that were used for isolation and purification of female germ line stem cells [13, 20]. DDX4/IFITM3-positive cells in endometriotic lesions also expressed, although weakly, OCT4 protein, a transcriptional factor necessary for maintaining the self-renewal and pluripotency phenotype of embryonic stem cells. Moreover, they expressed PCNA indicating self-renewal capacity and/or the mitotic activity of these cells. Thus, the finding of cells displaying germ cell-specific DDX4 and IFITM3 proteins suggests an ovarian origin, and as far as we could track in the literature this is the first evidence reported for ovarian-sourced cells in the endometrioma. In line with this, expression of pluripotency markers such as OCT4 and NANOG and the protoncogene *c-KIT* in stem-like cells in endometriosis have been described and related to the development of the disease and progression towards ovarian cancer [9, 21]. Nevertheless, the presence of ovarian germ line stem cells as contributors to endometriosis progression remains to be further studied through functional analysis in isolated DDX4/IFITM3-positive cells.

Alternatively to the prevailing theory of retrograde menstruation, it has been suggested that a small population of mesenchymal stem cells (eMSC) residing in the normal endometrial tissue may contribute to endometriosis progression [6, 7]. It is not possible to rule out that DDX4/IFITM3-positive cells are related to eMSC since weak expression of OCT4 may indicate differentiation towards stromal cells. However, the absence of DDX4/IFITM3-positive cells in normal endometrial tissue advocates in favour of an ovarian origin. Whatever the case may be, the identification of ovarian germ line and endometrial mesenchymal stem cells in endometriosis lesions adds up new ways in the understanding of this pathology whose pathogenesis still remains confusing. How stem cells may contribute to disease progression could be related to the hyperstrogenic environment that characterizes endometriosis, contributed by both systemic and locally synthesized estrogens [22]. Estrogen acts as a proliferation and differentiation agent not only in eutopic but also in ectopic endometrial cells [23]. In line with this, DDX4/IFITM3-positive cells were found to express ESR1 and revealed renewal activity by expressing PCNA.

Besides their essential role in germ line commitment, DDX4 and IFITM3 expression is also associated with other processes such as cell cycle progression [24, 25] and antiviral activity [26, 27]. IFITM3 is over expressed in different types of cancers, such as breast [28, 29], promoting epithelial-mesenchymal transition through the Wnt/β-catenin signaling. On the other hand, DDX4 reduces the expression of 14-3-3σ, which serves as a regulator for G2 checkpoint [25, 30]. Many reports have demonstrated the loss of expression of 14-3-3σ in human cancers [31, 32]. Furthermore, the relationship between endometriosis and cancer progression has been reported in endometriod epithelial ovarian cancer and ovarian clear cell carcinoma [33]. In this context, up regulation of DDX4 and IFITM3 proteins in ovarian-sourced stem cells, as well as OCT4 in eMSC, could correlate with the molecular pathway towards the malignant forms of the endometriosis lesion.

In conclusion, the data reported here identified DDX4- and IFITM3-expressing cells, two germ line-specific proteins, in ovarian endometriotic lesions, most likely arising from ovarian-sourced stem cells, suggesting a possible involvement of ovarian stem cells in the development of the endometrioma. This finding opens new doors towards deepening the understanding of this pathology and its progression.
